# Managed Competition in Healthcare (?) Comment on "Measuring Active Purchasing in Healthcare: Analysing Reallocations of Funds Between Providers to Evaluate Purchasing Systems Performance in the Netherlands"

**DOI:** 10.34172/ijhpm.9094

**Published:** 2025-08-02

**Authors:** Juraj Nemec

**Affiliations:** Faculty of Economics and Administration, Masaryk University Brno, Brno, Czechia.

**Keywords:** Managed Competition, Healthcare, Healthcare Reform

## Abstract

This article comments on the paper by Stadhouders et al titled "Measuring Active Purchasing in Healthcare: Analysing Reallocations of Funds Between Providers to Evaluate Purchasing Systems Performance in the Netherlands." Its main aim is to respond to the fact that the paper, without discussion, assumes that competitive reform stimulates the efficient allocation of funds. To achieve this goal, this article discusses existing knowledge related to the author’s assumption, highlighting that there is no uniform theory regarding the capacity of market forces to regulate healthcare markets. It also argues that market-based healthcare reforms may be very risky in countries with limited state regulatory capacity and widespread corruption.

## Introduction

 This article comments on the paper by Stadhouders et al titled “Measuring Active Purchasing in Healthcare: Analysing Reallocations of Funds Between Providers to Evaluate Purchasing Systems Performance in the Netherlands.” In this paper, the authors deliver additional information related to the long-term debate about the pros and cons of managed care—this debate is now approx. half a century-long but has not reached any definite conclusion. Any new, qualified, data-based input into this debate is critically important, both for academia and practice; therefore, the paper is relevant.

 The core findings of the paper are as follows: “Dutch managed competition and competitive purchaser reforms had no discernible effect on reallocations of funds between providers, casting doubt on the mechanisms advocated by managed competition and active purchasing to improve allocative efficiency” (p. 1).^1^

 Such a result is not any surprise for any well-informed health economics and policy expert – it is just necessary to say “thanks” for the additional evidence related to the topic in this technically well-written paper.

 Therefore, the goal of this commentary is not to discuss the methods and results of this paper but to reflect the following assumptions provided by the authors in the paper without any adequate discussion:

 “[*Theory predicts*] that competitive reform stimulates efficient allocation of funds. However, comparing different purchasing systems in the Netherlands reveals little evidence of elevated allocative activity, suggesting that competitive reforms may either have limited effect on healthcare efficiency, or currently unknown mechanisms are used to improve efficiency by competitive third-party payers [emphasis added]” (p. 2).^1^

 “[*Contrary to the theory of managed competition*], low reallocations of funds between providers were found in the competitive Dutch hospital sector, questioning the premise that managed competition improves allocative efficiency through selectively contracting high-quality providers [emphasis added]” (p. 2).^1^

 The fact that the authors automatically and without proper discussion of the already existing findings related to the potential of managed competition assume that managed competition should deliver results calls for discussion.

## Managed Care: Concept and Results

 Probably the most well-known advocate of managed competition is Alain Enthoven, who had already started to deal with the issue before 1980.^2^ In all his articles, he promotes the idea that managed competition stimulates better allocative efficiency and funds savings. Enthoven^5^ defines managed competition: “Managed competition is a purchasing strategy to obtain maximum value for money for employers and consumers. It uses competition rules derived from rational microeconomic principles to reward with more subscribers and revenue those health plans that do the best job of improving quality, cutting cost, and satisfying patients” (p. 29). Enthoven^3^ also tries to explain why managed competition should work: “Finally, competition is the way to achieve a system driven by the informed choices of consumers who are responsible for the cost consequences of their choices. A government-controlled system is driven by political forces” (p. 41).

 To react to the fact that the core institutional elements of managed competition in the United States (Health Maintenance Organisations and Preferred Provider Insurance) failed to help control the growing health expenditure in the United States, Enthoven^4^ argues as follows: Some say, “competition failed.” I say, “competition has not been tried.”

 At the same time, many authors evaluated the British National Health Service (NHS) marketisation-based experiment. The most well-known analyses are by Rudolf Klein (most were published with Patricia Day). Klein^5^ states: “The reforms of the NHS introduced in 1991 by Mrs Thatcher’s Conservative government were driven by much the same set of concerns and ideas that shaped the international debate vocabulary. In particular, they reflected the widely held belief that the best way of improving efficiency was to change the incentives to providers and that some form of marketlike competition was the best tool for achieving this aim” (p. 299). Klein^5^ also argues that similar reforms were discussed in many other countries (like Sweden, the Netherlands, and Germany, but only partially implemented). Most importantly, Klein^5^ is rather negative regarding short-term impacts of the market-based healthcare reforms in the UK: “The introduction of the reforms may have meant radical administrative changes, but their impact on the delivery of services turned out to be both extremely gradual and almost imperceptible. The shock to the system - the new demands made on healthcare professionals and managers by the introduction of the mimic market - did not translate into any immediate changes as far as consumers were concerned” (p. 309). He argues that it is impossible to document by data that both core objectives of the NHS (“to give patients, wherever they live in the UK, better healthcare and greater choice of services available” and “greater satisfaction and rewards for those working in the NHS who successfully respond to local needs and preferences”) were achieved.^5^

 Most later studies (the discussed article, too) provide evidence of the limited success of similar reforms worldwide. We cannot mention all of them; we need two examples. Andritsos and Tang^6^ studied the operational implications of competition in providing healthcare services in the context of national public healthcare systems in Europe. One of their questions was if the introduction of increased patient choice grants European patients the freedom to choose the country where they receive treatment. Their findings show that such freedom appears to materialise only in border regions, where the cost of crossing the border is low.

 Lieverdink analysed the same country as the authors of the discussed article – Netherlands, and he argues^7^: (The reform) … “has not led to sickness funds becoming powerful purchasers that forced hospitals and doctors to improve their efficiency. Rather, they compete for subscribers, become part of large insurance conglomerates, and market more supplementary options. Culturally, healthcare institutions have become more entrepreneurial, taken up more business concepts, and made the language of markets, products and consumer sovereignty more common. The impact of these changes on the healthcare system is still unknown, but they create pressure for more healthcare services, leaving the government with problems that equal those of the 1980s.”

 Both health economics and health policy publications try to explain why the chance of managed competition serving as the primary regulator of healthcare delivery is marginal. The health economics focus is mainly the “asymmetric information.” This asymmetry significantly limits the chance of healthcare markets achieving proper allocation of resources (See Arrow^8^). The unequal power relationship between experts (medical doctors) and clients (patients) which the former may exploit better information in their own interest is one of the core sources of the “market failure” in healthcare. The information asymmetry simply implies that patients cannot be “best judges of their needs,” as most market theories assume.

 The unequal power relationship between experts (medical doctors) and clients (patients), in which the former may exploit better information in their own interest, is one of the core sources of “market failure” in healthcare. The information asymmetry simply implies that patients cannot be “best judges of their needs,” as most market theories assume.

 Other frequently used arguments related to the limited chance of healthcare markets to regulate the demand and supply sides of healthcare delivery are local—regional monopolies (for example, it is more than difficult to close a low-performing hospital by top-down order), limited patient mobility, principal-agent relationships and some other arguments (we do not have space to go into the details).

 The public policy arguments are, for example, very nicely formulated by Saltman and Figueras^9^: “Experience to date suggests that health system reform if it is to be successful, must encompass considerably more than just cost-containment. Effective and sustainable reform also requires that healthcare services constitute a social good and that specific policy measures can increase health gain and the overall health status of the population.” Healthcare is probably the most complex public service, which means that the responsible actor – the state – should search for the best available combination of reform goals.

## Conclusions

 The discussion related to the potential of market forces (quasi-market) in managing healthcare systems has existed for approximately half a century and, without any doubt, will continue in the future. The purpose of this “never-ending story” is that the positions of experts (and subsequently also of governments) are normative. Some economists trust only to the free market (like Milton Friedman, Murray Newton Rothbard, Friedrich August von Hayek, and many others). Other economists (like Joseph Eugene Stiglitz or John Kenneth Galbraith) propose that markets fail. In such cases, the government may intervene or even more directly support government social and economic interventions.

 The core policy lessons connected with the topic are not formulated in the article, but it is obvious if we look at the existing knowledge and experience. With managed care (quasi-market), the role of the state changes – let us say that the state should switch from “producer” to “regulator.” However, even if the state as the original producer does not perform well, the chance that market-based reforms improve the situation in countries with limited government regulatory capacity and massive corruption is minimalistic. Thus, market-based reforms in healthcare may deliver certain positive outcomes in most developed countries if well implemented. However, less developed countries should seek stepwise reforms, with a focus on universal coverage, not to waste additional scarce resources.

## Ethical issues

 Not applicable.

## Conflicts of interest

 Author declares that he has no conflicts of interest.
